# Implementation of an AI-Driven Workflow for Daily Dose Reconstruction in Prostate Cancer Radiotherapy

**DOI:** 10.3390/cancers18111826

**Published:** 2026-06-02

**Authors:** Jessica Prunaretty, Tom Baudouin, Olivier Riou, David Azria, Pascal Fenoglietto

**Affiliations:** Institut du Cancer de Montpellier, 34090 Montpellier, France

**Keywords:** dose reconstruction, artificial intelligence, prostate cancer, Adaptbox, CBCT, automation

## Abstract

**Background/Objectives**: This study evaluated the daily delivered dose in prostate cancer patients using the automated artificial intelligence (AI)-based software Adaptbox (v2.3.2, Therapanacea). The aim was to assess target coverage and organ-at-risk (OAR) exposure. **Methods**: Twenty patients were included. All received 80 Gy in 40 fractions to the prostate and 56 Gy simultaneously to the seminal vesicles using two-arc VMAT on a TrueBeam STx, with daily CBCT for setup. For each fraction, CBCT images were imported into Adaptbox. A synthetic CT (sCT) was generated using a deep learning algorithm. OARs were automatically segmented, while targets were propagated from the planning CT (pCT) using rigid registration. Dose calculation was performed using Adaptbox’s collapse-cone algorithm. Dose parameters were extracted for each session and compared with planned values. **Results**: All 800 fractions were analyzed. The planning target volume (PTV) remained consistent with planning, with a maximum deviation of 0.1% for both PTVs. For the rectum, 78.38%, 77.75%, and 78.13% of fractions exceeded planned doses for V_70Gy_, V_76Gy_ and V_80Gy_, respectively. One patient had five consecutive fractions with >5% deviation across all rectal metrics. For the bladder, 52.34% of fractions exceeded the planned V_80Gy_, and two patients had ≥5 consecutive fractions with >5% deviation; however, this was attributed to contouring inaccuracies. **Conclusions**: This AI-based workflow enables reliable daily dose reconstruction and can identify clinically relevant OAR dose deviations that may support adaptive interventions, although accurate contouring remains essential.

## Simple Summary

This study explores the use of artificial intelligence to automatically estimate the radiation dose delivered to patients with prostate cancer during each treatment session. The goal is to better understand how the actual delivered dose may differ from the originally planned dose due to daily anatomical variations. Using specialized software that generates synthetic CT, outlines organs at risk, and calculates radiation dose automatically, 800 treatment sessions from 20 patients were analyzed. The results showed that the delivered dose to the target volumes remained consistent with the planned treatment. However, in some sessions, higher-than-expected doses were detected in nearby organs such as the rectum and the bladder. In two patients, these differences were mainly related to inaccuracies in the automatic bladder outlining. These findings suggest that artificial intelligence can help monitor daily radiation delivery, but expert visual verification remains important to ensure accurate and clinically reliable results.

## 1. Introduction

Radiotherapy is a key component in the management of prostate cancer across all prostatic groups [1,2]. Intensity-modulated radiation therapy (IMRT) and volumetric-modulated arc therapy (VMAT) currently represent the gold standard for prostate radiotherapy, as they provide superior dose conformity and are associated with reduced toxicity compared with three-dimensional conformal radiotherapy [3]. Despite these technological advances, daily anatomical variations remain a major source of uncertainty in prostate cancer radiotherapy [4]. Such variations may result in discrepancies between the planned and the actually delivered dose distributions [5].

Online adaptive radiotherapy (oART) has been increasingly implemented in clinical practice worldwide, as it allows treatment plans to be re-optimized based on patient-specific anatomical changes observed at each treatment fraction [6]. While several studies have reported dosimetric benefits in terms of improved target coverage and enhanced organ-at-risk sparing [7,8,9,10,11], clinical outcome data are still pending. Moreover, oART workflows remain time-consuming and require substantial human expertise as well as significant institutional resources, which currently limit their widespread adoption [12].

Cone-beam computed tomography (CBCT), routinely used for patient positioning, also provides valuable information on daily anatomical changes [13]. Consequently, CBCT-based dose reconstruction has been proposed as a potential tool to support clinical decision-making in ART, offering a more personalized approach without the full workload associated with oART. However, the direct use of CBCT for daily dose evaluation is limited by scatter-related artifacts and reconstruction algorithm constraints, leading to reduced image quality and compromised accuracy of CBCT-based dose calculations [14,15]. To overcome these limitations, several strategies have been developed to enable dose calculation on CBCT images [16], including CBCT calibration methods [17], Hounsfield Unit (HU) override techniques [18] and deformable image registration (DIR) approaches [19].

Among these, dose mapping based on image registration [20,21] relies on transferring the dose distribution from a reference image (i.e., planning CT) to a target image (i.e., CBCT). This approach accounts for interfraction anatomical changes and enables the evaluation of discrepancies between planned and delivered dose distributions, potentially triggering adaptive replanning when deviations become clinically significant. Nevertheless, the accuracy of dose mapping strongly depends on the reliability of deformation models used to establish voxel-to-voxel correspondence. This remains particularly challenging in regions with high anatomical variability, such as the pelvis.

Giacometti et al. [22] compared several CBCT-based dose calculation strategies and reported that, although all methods achieved acceptable dosimetric accuracy, dose mapping approaches were the most accurate. However, they also emphasized that these techniques require additional preprocessing steps, such as extending the CBCT field of view, applying density overrides, or using dedicated software for CT-to-CBCT deformable registration followed by DICOM generation. Although several commercial and open-source DIR solutions are now available, their robustness remains limited in complex clinical scenarios, such as prostate alignment [23].

More recently, deep learning-based approaches have been developed to improve CBCT-based dose workflows. Synthetic CT (sCT) generation from CBCT images was introduced to enhance image quality and overcome limitations of standard reconstruction. Kida et al. [24] were among the first to propose a deep convolutional neural network for CBCT enhancement in prostate cancer patients. Subsequent studies have demonstrated that deep learning methods can significantly improve image quality compared to DIR-based approaches [25,26,27], while also enabling more accurate dose calculations on sCT images. In parallel, artificial intelligence (AI)-based auto-segmentation has emerged as a key application in radiotherapy, facilitating faster, more consistent, and more automated dose reconstruction workflows [28].

Despite these advances, only a limited number of studies have reported clinically integrated workflows for fully automated CBCT-based daily dose reconstruction, and none have implemented an end-to-end deep learning solution [29,30,31,32,33,34]. Most previously published approaches still rely partially on deformable registration workflows or require substantial preprocessing and manual intervention, which may limit large-scale clinical implementation. Groot Koerkamp et al. [32] developed a fully automated CBCT-based dose evaluation workflow in breast cancer patients, demonstrating its ability to identify patients at risk of suboptimal treatment. Their approach combined DIR-based synthetic CT generation, contour propagation, and dose recalculation on daily anatomy. Similarly, Pesonen et al. [34] investigated a CBCT-based dose prediction workflow for adaptive radiotherapy in breast cancer, showing the potential of deep learning-based dose prediction and automated adaptation to improve target coverage and organ-at-risk sparing. However, these approaches still relied on deformable CT-based workflows rather than fully AI-driven CBCT pipelines.

The Adaptbox software (v2.3.2, Therapanacea) is an artificial intelligence-based platform that integrates CBCT-based synthetic CT generation, deep-learning U-Net 3D auto-segmentation, and accelerated dose calculation for dose reconstruction [35]. The aim of this study was not to develop a novel AI algorithm, but rather to evaluate the feasibility, robustness, and clinical relevance of implementing an AI-powered workflow for daily dose reconstruction in prostate cancer radiotherapy in routine clinical practice. In addition to workflow efficiency and processing time, this study investigated longitudinal delivered dose monitoring throughout treatment and explored the potential use of automated dose deviation analysis as a quality control strategy for adaptive radiotherapy workflows.

## 2. Materials and Methods

### 2.1. Patient Selection

Twenty patients with prostate cancer enrolled in the experimental arm of the RCMI-GI clinical trial [36] between August 2016 and September 2018 were retrospectively selected. This randomized phase II trial investigated late pelvic toxicity and quality of life after IMRT/IGRT with standard margins or with reduced margins enabled by real-time intrafraction motion tracking using the Calypso system (Varian) in patients with low- to intermediate-risk prostate cancer. All patients underwent planning CT scan (GE Optima CT580, General Electric Healthcare, Waukesha, WI, USA) with a 1.25 mm slice thickness. Clinical target volume (CTV) 1 comprised the prostate and seminal vesicles, while CTV2 included only the prostate. The proximal 2 cm of the seminal vesicles, measured from their attachment to the prostate in the cranio-caudal and lateral directions, were contoured. Although this approach is not standard for low-risk prostate cancer, inclusion of the proximal 2 cm of the seminal vesicles in CTV1 is consistent with longstanding local practice following the implementation of IMRT and established clinical trial protocols [37,38]. Planning target volume (PTV)2 was defined as a 3 mm expansion around the prostate, while PTV1 was the combination of PTV2 and an anisotropic margin around the seminal vesicles (1 cm cranially, 1 cm laterally, 0.3 cm anteriorly and 0.5 cm posteriorly) [39]. The rectum was delineated as a single solid organ extending 2 cm superiorly and inferiorly to CTV1 according to GETUG06 clinical trial [40]. The bladder was also contoured as a solid organ.

The prescription dose was 80 Gy in 40 fractions to the PTV2 and simultaneously 56 Gy to the PTV1. All treatment were delivered with VMAT using 2 arcs and a TrueBeam STx (Varian Medical Systems, Inc., Palo Alto, CA, USA). The optimization and the dose calculation were performed using Eclipse treatment planning system with the Photon Optimizer and AAA algorithms (Varian Medical Systems, Inc., Palo Alto, CA, USA), respectively. The dose constraints are detailed in Table 1.

A daily CBCT scan with soft tissue matching was performed on the tumor for patient positioning. Anatomical changes were visually assessed on the CBCT by the treating radiation therapists and real-time tracking was performed using the Calypso 4D Localization system (Varian Medical Systems, Inc., Palo Alto, CA, USA) [41].

### 2.2. Adaptbox Workflow

The Adaptbox software (v2.3.2, Therapanacea, Paris, France) is an AI-driven platform designed to automate offline adaptive radiotherapy workflow. The process involves importing the planning CT (pCT), including the associated structure set and treatment plan, as well as the daily cone-beam CT (CBCT) and the corresponding DICOM registration file generated during the treatment session.

First, an artificial intelligence-based synthetic CT (sCT) is generated from the CBCT using a CycleGAN architecture [42]. The model was trained on a multi-institutional dataset including CBCT images acquired from different linear accelerator manufacturers. The reported mean absolute error (MAE) of the generated sCT was 23.65 ± 10.09 HU across all anatomical regions.

All clinical target volumes (prostate and seminal vesicles) and PTVs are then propagated to the sCT using rigid registration. The use of rigid propagation for the prostate was based on previous work [43], in which it was preferred over deformable registration. Subsequently, organs at risk (OARs), including the bladder, rectum, and femoral heads, are automatically delineated on the sCT using a deep learning-based segmentation model. The performance of this auto-segmentation approach on CBCT-derived sCT images has been previously validated in a clinical study [44].

**Figure 1:**
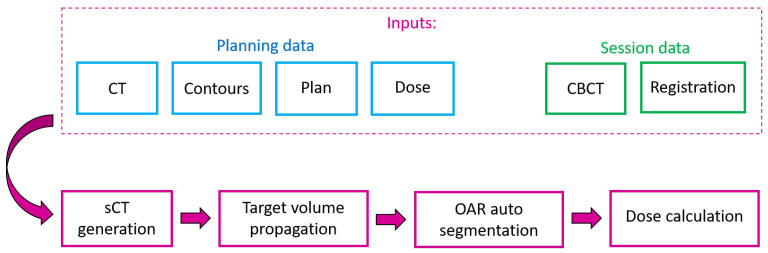
Schematic overview of the daily dose reconstruction workflow implemented in the Adaptbox software.

Finally, the daily delivered dose is computed by forward calculation of the reference treatment plan on the sCT and the propagated structures, using the proprietary collapsed-cone dose calculation engine. A previous study has validated the dose calculation engine for pelvic patients [35]. For consistency and fair comparison, the dose on the planning CT is also recalculated using the same dose engine. Differences between planned and delivered doses are evaluated using patient-specific dose–volume histogram (DVH) metrics. DVHs derived from the daily sCT and from the recalculated pCT can be exported in CSV format for further analysis. The overall workflow is illustrated in Figure 1.

### 2.3. Evaluation of the Delivered Dose

**Figure 2:**
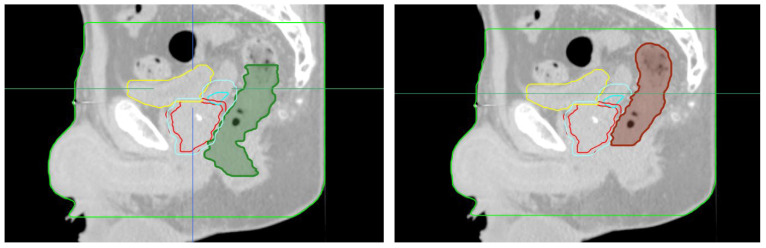
Example of rectum delineation difference between Adaptbox (**right** image and brown contour) and our reference (**left** image and green contour).

To ensure methodological consistency, the physician-delineated rectal contour on the pCT was not used, as the delineation rules differed from those applied by the auto-segmentation algorithm. Specifically, the 2 cm cranio-caudal extent defined in the clinical trial protocol was not respected by the automated contours. Figure 2 shows an example of rectum delineation difference between Adaptbox and our reference on the pCT. Consequently, the rectum was re-delineated on each pCT using the Adaptbox auto-segmentationn tool.

Dose metrics were evaluated according to the dose constraints summarized in Table 1. For each treatment fraction, the daily delivered dose was compared with the planned dose. A fixed threshold of 5% dose deviation was applied in this study as a pragmatic criterion. Consequently, when a dose deviation exceeding 5% was observed, the corresponding sessions were reviewed to verify the accuracy of auto-segmentation and dose distribution, and to identify potential causes for under or overdosage related to anatomical changes. Cumulative empirical triggers, defined as three or five consecutive sessions with a dose deviation exceeding 5%, were used as thresholds to detect patients potentially receiving suboptimal treatment.

## 3. Results

All 800 delivered fractions from 20 patients were analyzed. For a single fraction, the complete workflow—from sCT generation to DVH CSV file export—required an average processing time of 2 min and 40 s.

Table 2 compares the dose metrics for PTV2 and PTV1 between the planned and daily delivered doses. Target coverage (V_76Gy_ and V_53.2Gy_) remained consistent with the treatment plan for all patients, with a maximum deviation of 0.1% observed for both PTV2 and PTV1. Regarding the near-maximum dose (D_2%_) to PTV2, the difference between planned and delivered doses was less than 1%.

**Figure 3:**
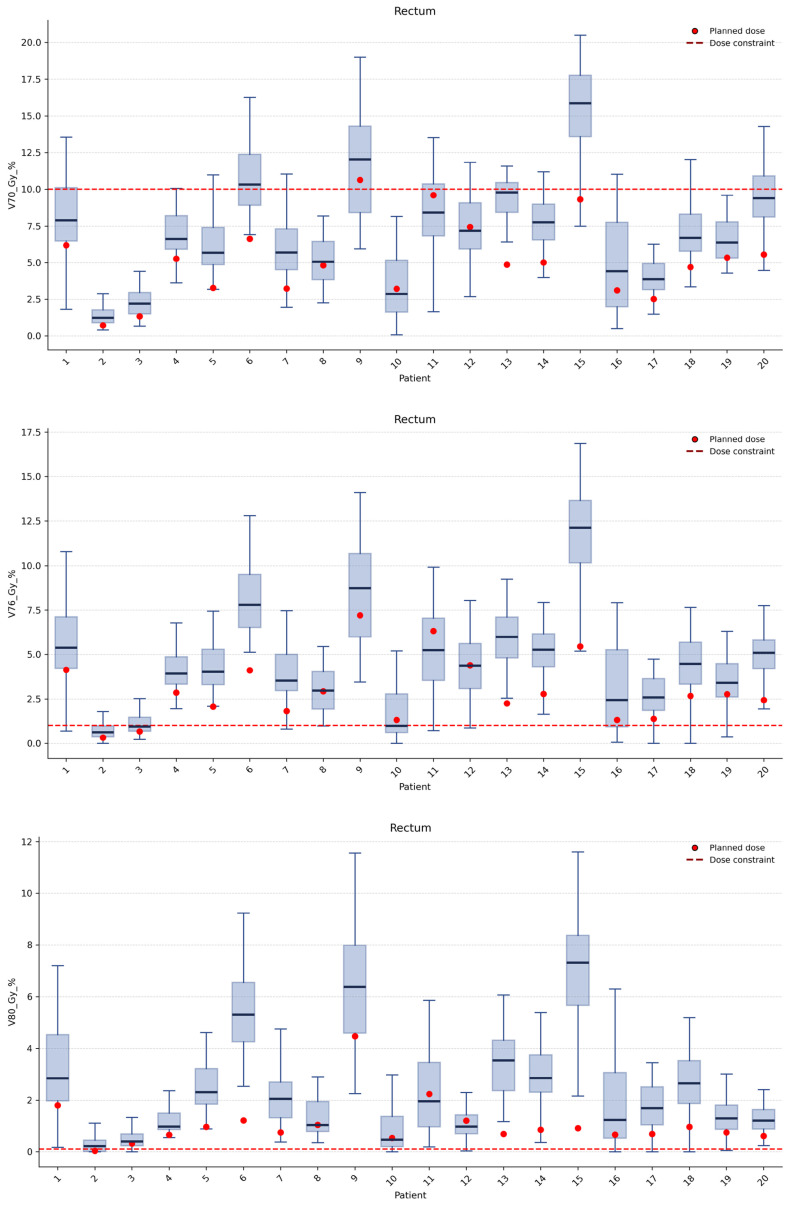
Comparison of the delivered dose (boxplot) and planned dose (point) for V_70Gy_, V_76Gy_ and V_80Gy_ of the rectum for each patient.

Figure 3 illustrates the variation in the daily delivered dose for each patient for rectal V_70Gy_, V_76Gy_, and V_80Gy_. Overall, most fractions delivered higher doses than initially planned. Specifically, 78.38%, 77.75%, and 78.13% of treatment sessions exceeded the planned dose for V_70Gy_, V_76Gy_, and V_80Gy_, respectively. Furthermore, deviations greater than 5% relative to the planned dose were observed in 13.91%, 9.99%, and 6.95% of sessions for V_70Gy_, V_76Gy_, and V_80Gy_, respectively.

Table 3 reports the number of patients exhibiting three or five consecutive fractions with deviations exceeding 5% from the planned dose for V_70Gy_, V_76Gy_, and V_80Gy_. Patient n° 15 was identified as having the highest number of fractions exceeding 5% from the planned dose.

**Figure 4:**
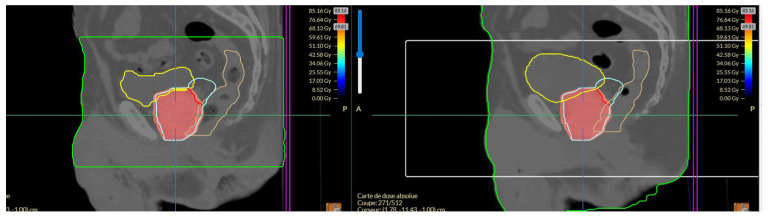
Comparison of 70 Gy isodose distribution between the planned dose (**left**) and the delivered dose (**right**) for the fraction 40 of the patient n° 15.

For this patient, a visual inspection of the sCTs and their associated contours was performed to identify potential causes of these deviations. Figure 4 illustrates the 70 Gy isodose distribution for patient n° 15, comparing the planned and delivered doses at fraction 40. Anatomical differences between sCT and pCT were identify and appropriately address within the workflow. In particular, an increased overlap between the rectum and the PTV was observed during treatment compared with the planning CT.

**Figure 5:**
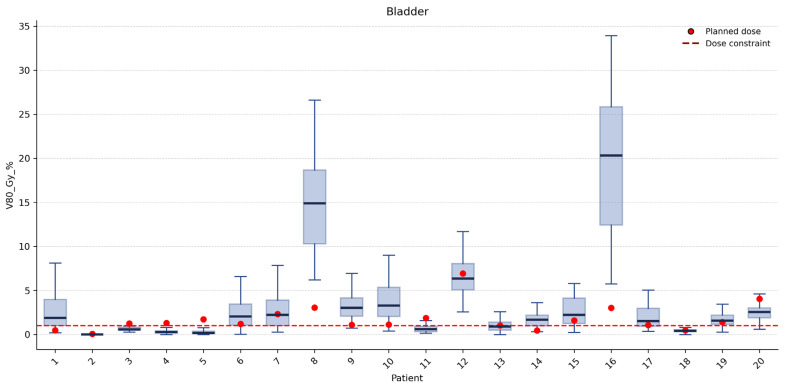
Comparison of the delivered dose (boxplot) and planned dose (point) for V_80Gy_ of the bladder for each patient.

Figure 5 illustrates the variation in daily delivered dose for each patient for bladder V_80Gy_ and compares with the planned dose. For the bladder, 52.34% of fractions delivered higher V_80Gy_ values than initially planned.

**Figure 6:**
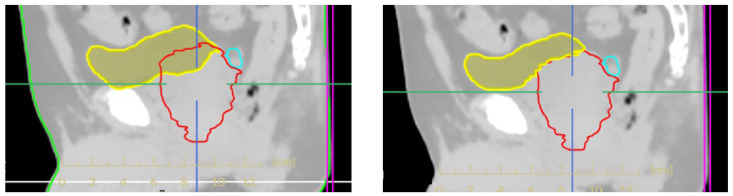
Comparison of bladder contour delineated by Adaptbox (**left**) and corrected (**right**) for patient 16.

Moreover, three patients (Nos. 6, 8, and 16) and two patients (Nos. 8 and 16) exhibited three and five consecutive fractions, respectively, with deviations exceeding 5% relative to the planned dose. Patients n° 8 and n° 16 showed the largest deviations compared with the planned dose. Therefore, the bladder contours for these two patients were carefully reviewed for each session. As shown in Figure 6 for patient n° 16, the junction between the bladder and the prostate was inconsistently delineated by Adaptbox.

**Figure 7:**
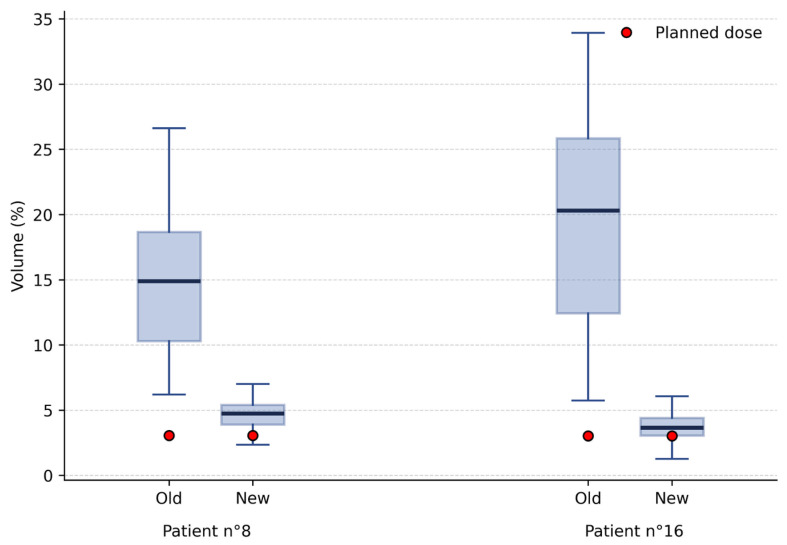
Comparison of V_80Gy_ results between the old contours (generated by Adaptbox) and new ones (corrected).

The bladder contours were subsequently corrected for these two patients. Figure 7 compares the V_80Gy_ values obtained using the Adaptbox contours and the corrected contours. V_80Gy_ values were significantly lower with the corrected contours and were within a 5% deviation from the planned dose.

## 4. Discussion

This study demonstrated a feasible automated AI-driven workflow for daily dose reconstruction based on synthetic CT. The workflow was evaluated on a dataset of 20 prostate cancer patients and provided fast and robust results, with an average processing time of 2 min and 40 s per fraction.

The objective of this work was not to develop a new AI segmentation or sCT generation algorithm, but rather to clinically evaluate and validate the integration of a complete end-to-end workflow for automated daily dose monitoring in routine practice. In this context, the proposed workflow combines automated synthetic CT generation, AI-based contouring, daily dose recalculation, and monitoring of delivered dose deviations throughout treatment. This translational and workflow-oriented evaluation represents an important step toward practical implementation of automated adaptive radiotherapy quality control strategies.

Target coverage was maintained throughout treatment for all patients and remained consistent with the planned doses. The use of rigid propagation for the target volumes can be critical; however, its application in this context was based on previous studies and is consistent with the characteristics of the selected patient cohort. Although the prostate is susceptible to deformation due to surrounding structures such as the rectum, the patients included in this study were part of the experimental arm of the RCMI-GI trial. In this setting, three intraprostatic transponders were implanted, and daily patient positioning using CBCT was guided by these markers. This strategy ensured highly accurate prostate alignment and allowed for minimization and quantification of residual deformation uncertainties.

Regarding rectal dose metrics, more than 70% of treatment fractions exceeded the planned values. However, only one patient exhibited five consecutive sessions with a delivered dose deviation greater than 5% relative to the plan across all metrics of the rectum. The 5% threshold was initially selected as a pragmatic and conservative value to illustrate the proposed workflow, rather than as a statistically optimized criterion. The primary objective of this study was to demonstrate feasibility and clinical relevance rather than to establish optimized tolerance limits. A more rigorous approach could involve metric-specific thresholds derived from retrospective statistical analyses of dose variability, such as population-based distributions or confidence intervals. This would allow the definition of clinically meaningful tolerance limits and further strengthen the robustness of the proposed quality control framework.

Moreover, while acceptability thresholds for each structure and associated metric are already user-defined within the software, the implementation of a criterion based on consecutive fractions exceeding the defined deviation (5% in this study) would further strengthen the system. Such an approach would enable the detection of systematic trends rather than isolated fluctuations and could serve as a robust automated quality control mechanism, supporting timely intervention by the physicist or physician in replanning decisions and potentially improving clinical care.

For the bladder, two patients had five consecutive fractions in which the deviation exceeded 5% relative to the planned dose. However, the results for two of these patients were attributed to inaccuracies in the bladder autocontouring generated by Adaptbox. In such cases, manual contour correction requires re-running the workflow even after dose computation has been completed. On a per-fraction basis, this correction typically adds approximately 2–3 min to the overall processing time. These findings highlight that, despite a high level of automation, visual verification of contours and dose metrics remains necessary to ensure reliability and clinical relevance.

Previous studies have demonstrated a stronger correlation between delivered dose and clinical outcomes [45,46]. Consequently, this automated workflow would provide a new dataset based on the daily delivered dose, rather than on the dose planned on CT acquired a few days or weeks before treatment. As a result, robust dose–toxicity models based on delivered dose could be established, improving the prediction of treatment-related toxicity and facilitating the implementation of adaptive radiotherapy strategies.

In this study, the delivered dose was evaluated on a per-fraction basis rather than using accumulated delivered dose. This approach represents a conservative strategy, as it highlights potential worst-case deviations while avoiding the additional uncertainties associated with dose accumulation [20,47]. In the pelvic region, dose accumulation typically relies on deformable image registration, which may introduce uncertainties due to substantial interfraction anatomical variations of the bladder and rectum. Consequently, evaluating dose deviations on a per-fraction basis provides a robust and straightforward framework for identifying clinically relevant deviations during treatment.

Finally, the Adaptbox workflow relies primarily on AI-based segmentation for most structures, in contrast to other systems that are based on deformable registration, such as ChartCheck Adaptive (Radformation) or ART.1 (SeeTreat). While automatic segmentation has demonstrated advantages in terms of robustness and accuracy, it does not rely on the initial clinician-defined contours. As a result, discrepancies may arise when institutions follow specific contouring guidelines. This was observed in the present study for rectum delineation, as the institutional practice based on the GETUG guidelines consists of contouring the rectum 2 cm above and below the CTV. These considerations highlight the importance of adapting or validating automated workflows within the context of local contouring practices. Furthermore, such inconsistencies may increase clinician workload due to secondary verification. This AI-based workflow could therefore benefit from customizable parameters or rule-based definitions to accommodate institution-specific guidelines. For example, the rectal contour could be defined as a single solid organ extending 2 cm superiorly and inferiorly to CTV1, while seminal vesicles could be defined as the proximal 2 cm from their attachment to the prostate in the cranio-caudal and lateral directions.

## 5. Conclusions

This study demonstrates the feasibility and clinical relevance of an automated AI-driven workflow for daily dose reconstruction based on synthetic CT, providing fast, robust, and clinically consistent results. While the 5% deviation threshold serves as a pragmatic starting point, future work should focus on refining metric-specific criteria and incorporating trends across consecutive fractions to improve clinical decision-making.

Despite high automation, visual verification and adaptation to local contouring practices remain essential.

## Figures and Tables

**Table 1:** Dose constraints in the RCMIGI clinical trial.

PTV2	V_76Gy_ ≥ 99.5–99% V_72Gy_ ≥ 90% D_2%_ ≤ 107%
PTV1	V_53.2Gy_ ≥ 95%
Rectum	V_76Gy_ ≤ 1% V_70Gy_ ≤ 10% V_80Gy_ ≤ 0.1%
Bladder	V_80Gy_ ≤ 1%

**Table 2:** Comparison of dose metrics between planned and delivered dose for PTV2 and PTV1.

		Planned	Delivered
PTV2	V_76Gy_ (%)	99.22 ± 0.11	99.30 ± 0.21
	D_2%_ (%)	107.17 ± 1.52	108.05 ± 1.64
PTV1	V_53.2Gy_ (%)	99.58 ± 0.27	99.57 ± 0.39

**Table 3:** Number of patients with 3 and 5 consecutive fractions showing >5% deviation from the planned dose for V_70Gy_, and V_76Gy_ of the rectum.

	V_70Gy_		V_76Gy_		V_80Gy_	
	3 Consecutive Sessions	5 Consecutive Sessions	3 Consecutive Sessions	5 Consecutive Sessions	3 Consecutive Sessions	5 Consecutive Sessions
Number of patients	4	2	3	1	2	1
Patient ID	n° 6–13–15–20	n° 15–20	n° 6–13–15	n° 15	n° 6–15	n° 15

## Data Availability

The datasets used and/or analyzed during the current study are available from the corresponding author on reasonable request.
